# Large-area perovskite solar cells – a review of recent progress and issues

**DOI:** 10.1039/c8ra00384j

**Published:** 2018-03-14

**Authors:** Yichuan Chen, Linrui Zhang, Yongzhe Zhang, Hongli Gao, Hui Yan

**Affiliations:** College of Materials Science and Engineering, Beijing University of Technology Beijing 100124 China yzzhang@bjut.edu.cn hyan@bjut.edu.cn; School of Mechanical and Electrical Engineering, Jingdezhen Ceramic Institute Jingdezhen Jiangxi 333403 China

## Abstract

In recent years, perovskite solar cells (PSCs) have attracted great attention in the photovoltaic research field, because of their high-efficiency (certified 22.1%) and low-cost. In this review paper, we briefly introduce the history of efficiency development for PSCs, and discuss some of the major problems for large-area (≥1 cm^2^) PSC devices. In addition, we summarize the recent progress in the aspects of fabrication methods for large-area perovskite films, and improving the efficiency and stability of the large-area PSC devices. Finally, we give a short summary and outlook of large-area PSC devices. This article is mainly organized into three parts. The first part focuses on the main fabricating technologies for large-area perovskite films. The second section discusses some methods that are used to improve the efficiency of PSCs. In the last part, different approaches are used to improve the stability of PSCs.

## Abstract 2

In this review, we summarize the recent progress in the aspects of the fabrication methods for large-area perovskite films, improving the efficiency and stability of the large-area PSC devices.

## Graphical abstract

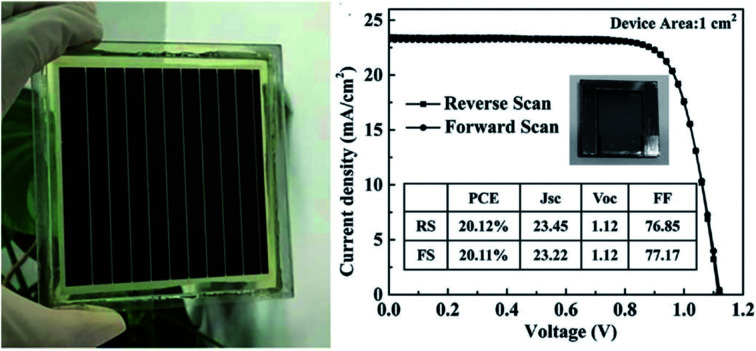

## 1. Introduction

Due to the growing population, the global energy demand is increasing year by year. Moreover, the global energy demand is predicted to double by 2050.^1,2^ Thus, the development of renewable energy becomes an imminent requirement, such as water energy, wind energy, and solar energy. The photovoltaic power generation capacity is installed to be 303 GW and increased 75 GW in 2016. In 2016, photovoltaic power generation accounted for only 1.5% of the world's total electricity generation. So high performance, long-term stability, low cost and environmental friendly solar cells become the focus of current energy research.

PSCs have attracted great attention in photovoltaic research in recent years, because of their high-efficiency (certified 22.1%)^3^ and low-cost. Meanwhile, organic–inorganic perovskites have a high optical absorption coefficient^4^ and the diffusion lengths exceed 1 μm for electrons and holes.^5^ So, organic–inorganic perovskite is an ideal absorber material for solar cells,^6–19^ photodetectors,^20–22^ light-emitting diodes,^23–26^*etc.*

**Fig. 1:**
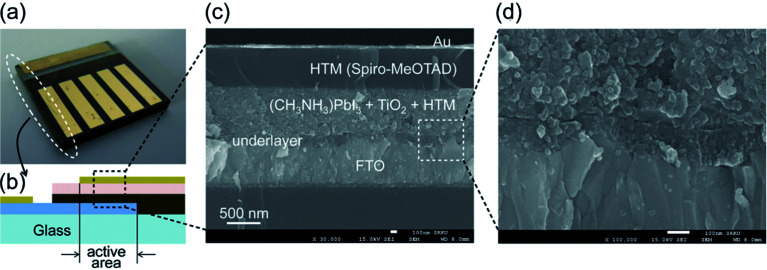
(a) Real solid-state device. (b) Cross-sectional structure of the device. (c) Cross-sectional SEM image of the device. (d) Active layer–underlayer–FTO interfacial junction structure.^27^

**Fig. 2:**
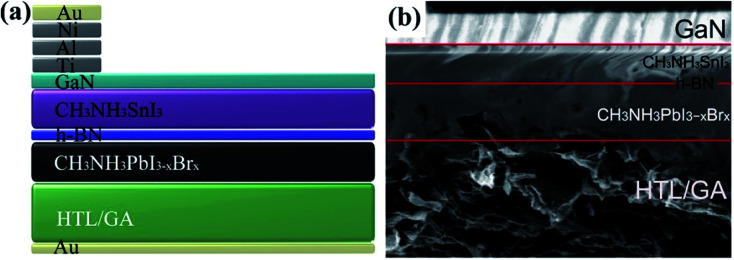
(a) Cross-sectional schematic. (b) SEM images of perovskite cell with integral monolayer h-BN and graphene aerogel.^30^

In recent years, hybrid metal halide perovskite materials have revolutionized the field of photovoltaics materials research, due to the power conversion efficiency (PCE) of PSC devices having been rapidly improved, from the point 3.8% in 2009,^6^ up to 22.6% in 2017 (ref. 3) (certified 22.1%).^3^ It attracted attention of researchers working on various photovoltaic technologies, especially dye solar cells (DSCs) and organic photovoltaic (OPV) with emphasis on better efficiency. In 2009, T. Miyasaka *et al.*^6^ has creatively made CH_3_NH_3_PbBr_3_/TiO_2_-based and CH_3_NH_3_PbI_3_/TiO_2_-based DSCs, the PCE of the cells is 3.13% and 3.81%, respectively. The PSCs attracted researchers' attention then happened in 2012, when M. Grätzel and N. G. Park *et al.*^27^ made PSCs device using perovskite films as the photoactive absorber layer, the mp-TiO_2_ and spiro-MeOTAD were used as the electron transport layer (ETL) and hole transport layer (HTL), respectively (Fig. 1), achieving the PCE of 9.7%. In 2013, M. Z. Liu, M. B. Johnston and H. J. Snaith^8^ fabricated planar heterojunction PSCs *via* vapor deposition, and the efficiency of the PSCs device is up to 15.4%. The yttrium (Y) doping the TiO_2_ (ETL) improves the electron transport channel in the PSCs device, and increase its carrier concentration and modify the ITO electrode to reduce its work function. These changes achieved a PCE of 19.3%.^28^ In 2015, S. I. Seok *et al.*^29^ attained an efficiency of PSCs up to 20.1%. In 2016, A. Zettl *et al.*^30^ made an architecture of GaN/CH_3_NH_3_SnI_3_/monolayer h-BN/CH_3_NH_3_PbI_3−*x*_Br_*x*_/HTL and graphene aerogel/Au (Fig. 2). The graded bandgap PSCs demonstrated with PCE averaging 18.4%, with a best of 21.7%. Other researchers, E. H. Sargent *et al.*^31^ (2017) achieved the certified efficiencies of 20.1% *via* contact-passivation strategy, retaining 90% (97% after dark recovery) of their initial PCE after 500 hours of continuous room-temperature. Meanwhile, E. K. Kim, J. H. Noh, and S. I. Seok *et al.*^3^ reported that the introduction of additional iodide ions into the organic cation solution, that was used to form the perovskite layers through an intramolecular exchanging process and decrease the concentration of deep-level defects. The certified PCE of PSCs attained 22.1%.^3^

In addition, high efficiency PSCs devices include not only small devices, but also larger cells. A PSCs device with area of large-area (≥1 cm^2^) and maximum PCE of 20.5% (certified 19.7%) has been reported.^3^Table 1 shows some results for large-area PSCs have been reported in the literatures.

**Table 1:** Summary of large-area perovskite solar cells

PSCs configuration	Cells area (cm^2^)	Active area (cm^2^)	PCE (%)	Ref.
ITO/PEDOT:PSS/MAPbI_3_/PCBM/C_60_/BCP/Al	1	1	12.2	32
FTO/TiO_2_/MAPbI_3_/spiro-OMeTAD/Au	1	1	11.7	33
ITO/HTL/PFN/CH_3_NH_3_PbI_3_/PCBM/Al	1	1	17.04	34
FTO/TiO_2_/(FAPbI_3_)_0.85_(MAPbBr_3_)_0.15_/spiro-OMeTAD/Au	1	1	18.32	35
FTO/c-TiO_2_/me-TiO_2_/MAPbI_3_/spiro-OMeTAD/Au	1	1	19.3	36
FTO/bl-TiO_2_/mp-TiO_2_/perovskite/spiro-OMeTAD/Au	1	1	19.6 (certified)	37
FTO/TiO_2_/me-TiO_2_:perovskite/perovskite/PTAA/Au	1	1	19.7 (certified)	3
ITO/SnO_2_/(FAPbI_3_)_1−*x*_(MAPbBr_3_)_*x*_/spiro-OMeTAD/Au	1	1	20.1 (certified)	38
ITO/TiO_2_/CH_3_NH_3_PbI_3−*x*_Cl_*x*_/spiro-OMeTAD/Au	4	1	13.6	39
FTO/c-TiO_2_/me-TiO_2_/perovskite/spiro-OMeTAD/Au	1.01	1.01	16.61	40
FTO/NiMgLiO/MAPbI_3_/PCBM/Ti(Nb)O_*x*_/Ag	1.02	1.02	16.2 (15 certified)	41
Anode/HEL/perovskite/gradient interlayer/ETL/cathode	1.022	1.022	18.21 (certified)	42
FTO/c-TiO_2_/me-TiO_2_/perovskite/spiro-OMeTAD/Au	1.05	1.05	15.89	43
FTO/ZnO/MAPbI_3_/spiro-OMeTAD/Au	1.10	1.10	3.08	44
FTO/TiO_2_–Cl/FA_0.85_MA_0.15_PbI_2.55_Br_0.45_/spiro-OMeTAD/Au	1.10	1.10	19.5 (certified)	31
SAM/PC_61_BM/MAPbI_3_/PTAA/Ag	1.20	1.20	15.98	45
ITO/PEDOT:PSS/MAPbI_3_/PCBM/C_60_/BCP/Al	64.0	1.50	6.0	32
ITO/PEDOT:PSS/(PEI)_2_(MA)_*n*−1_Pb_*n*_I_3*n*+1_/PCBM/LiF/Ag	2.32	2.32	8.77	46
SAM/PC_61_BM/MAPbI_3_/PTAA/Ag	5.04	5.04	12.79	45
FTO/c-TiO_2_/TiO_2_ or MAPbI_3_/spiro-OMeTAD/Au	10.1	10.1	10.4	47
FTO/c-TiO_2_/me-TiO_2_/MAPbI_3_/spiro-OMeTAD/Au	36.0	36.0	15.7 (12.1 certified)	36
FTO/c-TiO_2_/TiO_2_ or MAPbI_3_/spiro-OMeTAD/Au	100	100	4.3	47

But, for large-area PSCs device, it still has some issues need to be solved, namely fabrication, stability, hysteresis, fabrication cost and environmental concerns. Such as, the continuous fabrication of cracks-free and pinholes-free the perovskite and the selective carrier extraction layers films is difficulty with large-area PSCs devices. The dilemma with optimizing such charge carrier extraction layers in solar cells is that the film should be thin to minimize resistive losses, while at the same time, it should cover the entire collector area in a contiguous and uniform manner.^48^ In the large-area PSCs device, surfaces, bulk defects and interfaces introduce recombination centers that lead to fast nonradiative losses,^49^ and interface losses, which lead to the *V*_oc_, *J*_sc_ and fill factor (FF) decrease. Meanwhile, the perovskite material is easily thermal decomposition and hydrodecomposition, that leads to the lack of stability for PSCs device. The poor stability of the perovskite materials and devices is a big challenge, which hinder the PSCs device could be transferred from the laboratory to industry and outdoor applications. Thus, for large-area PSCs device, the major challenges relate to the improving efficiency and keeping the stability of the device. In this review paper, giving an update of the PSCs field, briefly, introducing the history of PSCs and then focus on the key progress of the fabrication, improving the efficiency and the stability of the large-area PSCs device.

## 2. Perovskite structure and typical PSCs structure

### 2.1 Perovskite structure and characteristics

**Fig. 3:**
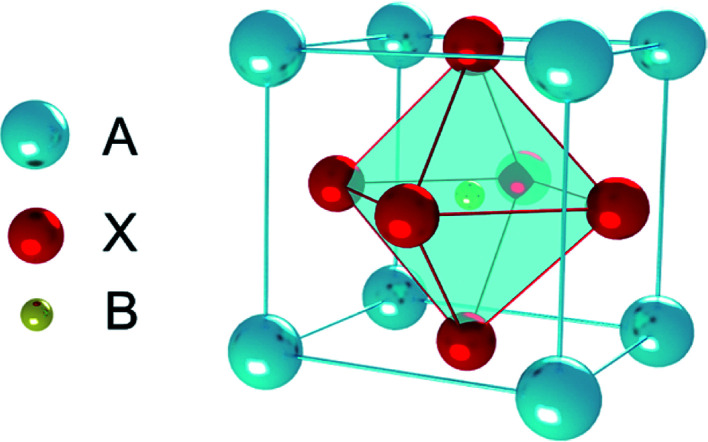
ABX_3_ perovskite structure.

Perovskite was discovered in 1839, which originally referred to a kind of ceramic oxides with the general molecular formula ABX_3_.^1^ Recently, PSCs absorber layer is mainly organic–inorganic perovskite layer, the general molecular formula is also ABX_3_ (Fig. 3), where A is an organic cation (*i.e.* CH_3_NH_3_^+^, NH_2_CH=NH_2_^+^, CH_3_CH_2_NH_3_^+^), B is metal cation (*i.e.* Pb^2+^, Sn^2+^, Ge^2+^) and X is halogen anion (*i.e.* F^−^, Cl^−^, Br^−^, I^−^), are the most relevant ones for PSCs.

The perovskite arrangement is approximated on its geometric tolerance factor (*t*),1
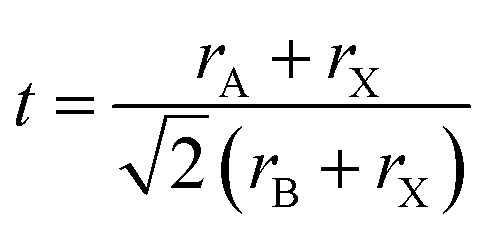
where *r*_A_, *r*_B_ and *r*_X_ are the efficient ionic radius for A, B and X ions, respectively. When the *t* = 1.0, the perovskite is a perfect cubic perovskite.^50^ However, octahedral distortion is assessed when *t* < 1, which influences electronic characteristics.^51^ For alkali metal halide perovskite, formability is anticipated for 0.813 < *t* < 1.107.^50,51^ In Table 2, the *r*_A_ in APbX_3_ (X = Cl, Br, I) perovskite has been calculated for *t* = 0.8 and *t* = 1 based on effective ionic radii.^51^ As the tolerance of CH_3_NH_3_PbI_3_ (MAPbI_3_) is 0.83, in this manner, the deviation from a perfect cubic structure is likely to happen.^50,51^

**Table 2:** Estimation of A cation radii in APbX_3_

*r*_Pb_ a	X^a^	*r*_A_ b for *t* = 0.8	*r*_A_ b for *t* = 1.0
Pb^2+^ (1.19 Å)	Cl^−^ (*r*_Cl_ = 1.81 Å)	1.58 Å	2.43 Å
	Br^−^ (*r*_Br_ = 1.96 Å)	1.60 Å	2.50 Å
	I^−^ (*r*_I_ = 2.20 Å)	1.64 Å	2.59 Å

a Effective ionic radii for coordination number of 6.

b
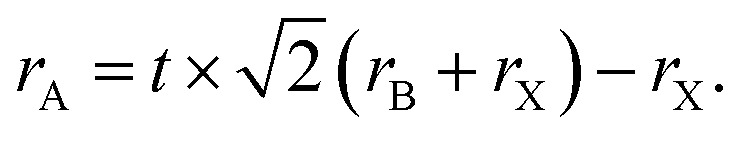
 ^50,51^

In the visible range, for the MAPbI_3_, the effective absorption coefficient is around 1.0 × 10^5^ (mol L^−1^)^−1^ cm^−1^ at 550 nm,^4,52^ when the thickness of perovskite films range is 500–600 nm, it can absorb complete light in films. Meanwhile, organic–inorganic perovskite exhibits better charge transfer characteristics. H. J. Snaith *et al.*^5^ reported the diffusion lengths (*L*_D_) of the electrons and holes in MAPbI_3_ and MAPbI_3−*x*_Cl_*x*_, the *L*_D_ of MAPbI_3_ is 130 nm (electrons) and 100 nm (holes) and this of MAPbI_3−*x*_Cl_*x*_ is 1100 nm (electrons) and 1200 nm (holes), respectively.^5^ So, the organic–inorganic perovskite is an ideal absorber layer material for solar cells.

### 2.2 Typical PSCs structure

**Fig. 4:**
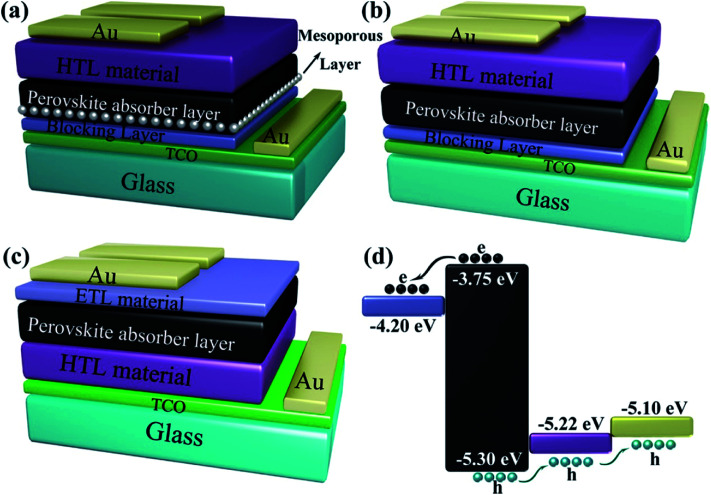
Different structural configurations of PSCs, (a) mesoporous structure, (b) planar heterojunction structure, (c) inverted planar heterojunction structure and (d) schematic of electron and hole transportation.

Some of the typical structures of PSCs are shown in Fig. 4. The typical PSCs structures include the mesoporous structure (Fig. 4(a)), the planar heterojunction structure (Fig. 4(b)) and the inverted planar heterojunction structure (Fig. 4(c)). PSCs with regular configuration is transparent conductive oxide (TCO)/blocking layer (electron transport layer (ETL))/perovskite absorber layer/hole transport layer (HTL) material/gold (Au). The widely accepted a simplified operation principle of PSCs is presented as: perovskite absorber layer absorbs light and generates charges while the light on the PSCs. The electrons and holes pairs are created by the thermal energy, which diffuse and get separate through electron and hole selective contacts, respectively (Fig. 4(d)).^53^ Once electrons and holes are present at the cathode and anode, respectively, external load can be powered by connecting a circuit through it.

**Fig. 5:**
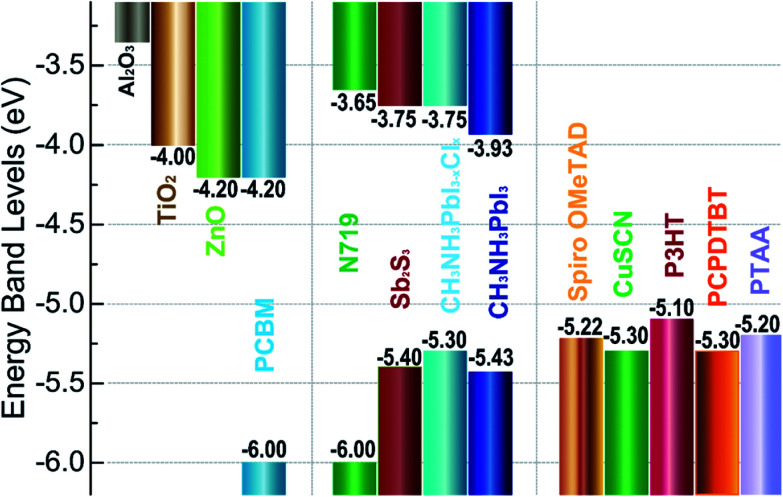
Energy levels for some materials of ETL (left), absorbers (middle) and HTL (right) in solar cells.

TiO_2_ is the most common ETL material,^3,6,7,54^ meanwhile, other ETL materials have been used to attain over 10% efficiencies (*e.g.* ZnO,^13,55,56^ SnO_2_,^57–59^ PCBM,^60–65^ LBSO,^66^*etc.*). Spiro-OMeTAD is the widely used HTL material,^35,57,67^ the certified PCE of 22.1% in small cells.^3^ Meanwhile, other HTL materials have been used to achieve over 10% efficiencies (*e.g.* PTAA,^29,66,68^ P3HT,^69–71^ PEDOT:PSS,^60,61,72^ CuSCN,^73,74^ triazine-Th-OMeTPA,^75^ PVCz-OMeDAD,^76^ OMeTPA-BDT,^77^ NiO_*x*_,^56,64,78,79^ CuGaO_2_,^54^ X26, ^80^ X36,^80^ NiMgLiO^41^*etc.*). Carbon materials,^81–85^ aluminum,^34,56,61,62^ silver,^64,65,86^ and gold^87–90^ have been used as electrode.^53^Fig. 5 shows the energy levels for some commonly used ETL materials, HTL materials and absorbers materials.

## 3. Large-area (≥1 cm^2^) perovskite films fabricating technologies

The continuous fabrication of cracks- and pinholes-free the perovskite films and the selective carrier extraction layers films is difficulty for the large-area PSCs devices. So, some researchers have reported many fabrication methods to improve the quality of the large-area perovskite films.

### 3.1 Spin-coating and vacuum flash-assisted solution process (VASP)

**Fig. 6:**
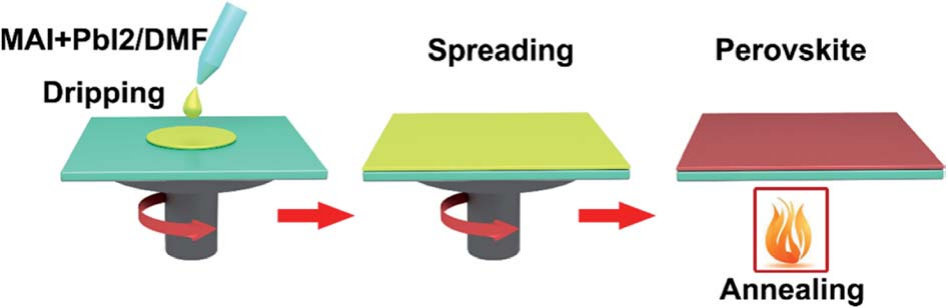
One-step deposited perovskite films.

Spin-coating has been widely used to fabricate the large-area perovskite films.^16,35,39–41,45,91^ The main advantage of the spin-coating method is to deposit thin films with well-defined the composition of chemical elements and the film thicknesses. Spin-coating includes one step spin-coating and two step spin-coating. One step spin-coating, briefly, methyl ammonium iodide (MAI) and lead iodide (PbI_2_) powders are mixed and dissolved in *N*,*N*-dimethylformamide (DMF) or dimethyl sulfoxide (DMSO), the mixed solution is spun on a TCO substrate and then annealed, attaining the perovskite films (Fig. 6). In 2015, M. Grätzel and L. Y. Han *et al.*^41^ prepared perovskite absorber films *via* one step spin-coating, they achieved large-area PSCs with an active area 1.02 cm^2^ that had a PCE > 15% (certified 15%). In 2016, W. Qiu and P. Heremans *et al.*^39^ achieved large-area PSCs with 4 cm^2^ aperture area and an active area of 1 cm^2^, that had a PCE of 13.6%.

**Fig. 7:**
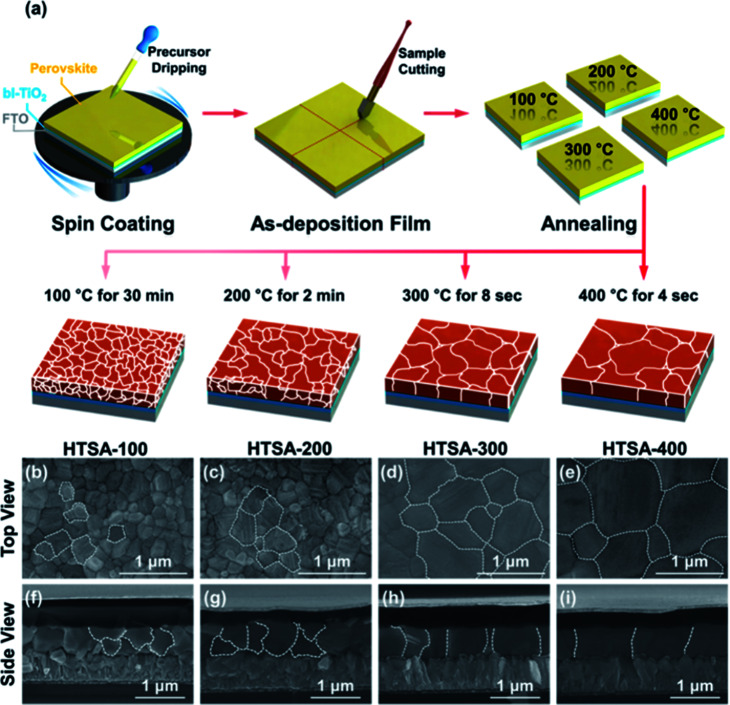
(a) Schematic illustration of the annealing processes. (b–e) Surface SEM images (top view) of the perovskite films. (f–i) Their cross-sectional images (side view), respectively.^35^

**Fig. 8:**
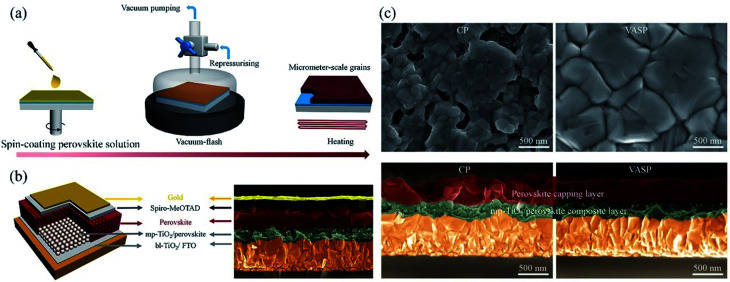
(a) Schematic illustration of nucleation and crystallization procedures during the formation of perovskite film *via* VASP. (b) Schematic illustration of the PSCs configuration and SEM image. (c) Surface and cross-sectional SEM images of the perovskite films fabricated by the conventional process (CP) and VASP.^37^

Aiming at uncovered pinhole areas derive from large perovskite grains, M. J. Kim and G. H. Kim *et al.*^35^ also developed one step spin-coating, and using high-temperature short-time annealing (HTSA) process (Fig. 7(a)), achieving the perovskite grains with sizes more than 1 μm without pinhole (Fig. 7(d, e, h, i)). In addition, the VASP was used to fabricate perovskite film (Fig. 8(a)), the sizes of perovskite grains were between 400 and 1000 nm (Fig. 8(c)), which covered the TiO_2_ layer.^37^

**Fig. 9:**
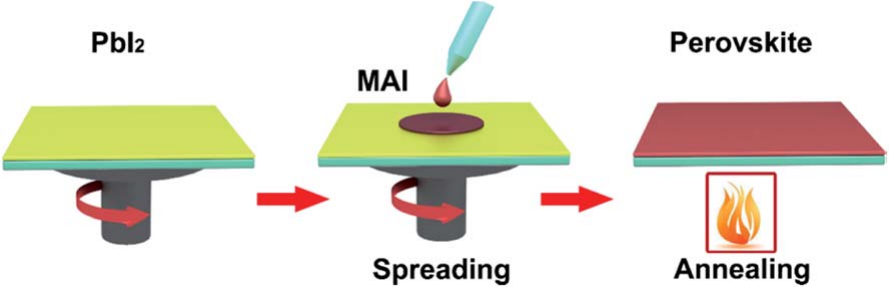
Two-step deposited perovskite films.

Two-step spin coating, briefly, MAI and PbI_2_ powders are dissolved in DMF or DMSO, respectively.^16^ First, the PbI_2_ solution is spun coating on a TCO substrate and then annealing, achieving the PbI_2_ films. Second, the MAI solution is spun coating on PbI_2_ films and then annealing, achieving the perovskite films (Fig. 9). In 2016, C. Chang *et al.*^45^ prepared perovskite absorber films with two step spin-coating, they achieved large-area PSCs with an active area 1.2 cm^2^ that had a PCE of 16.2%. In 2017, E. K. Kim, J. H. Noh and S. I. Seok *et al.*^3^ achieved large-area PSCs with an active area 1 cm^2^ that had a certified PCE of 19.7%. In 2017, X. W. Zhang and J. B. You *et al.*^38^ have adopted two-step spin-coating method to fabricate the (FAPbI_3_)_1−*x*_(MAPbBr_3_)_*x*_ films and configure n–i–p planar structure PSCs with an active area 1 cm^2^ that has a PCE of 20.1%.

### 3.2 Vapor deposition

**Fig. 10:**
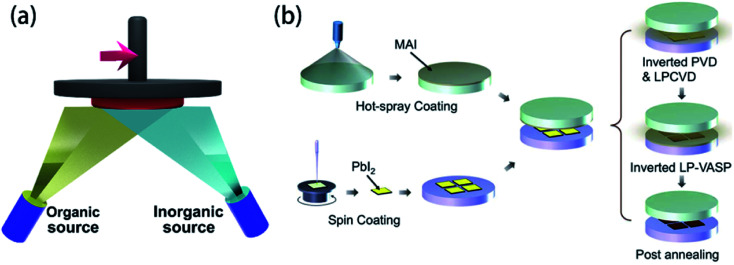
(a) Schematic illustration of double source co-evaporation. (b) Schematic of vapor–solid reaction method.^32^

Comparing to the fabrication of the PSCs device with the spin-coating technology, vapor deposition technology offers a very superior device and superior performance (Fig. 10(a)). The vapor deposition includes dual-source evaporation technology,^8^ vapor–solid reaction,^32^ and vapor-assisted method,^50^*etc.* For dual-source co-evaporation technology, it is that PbI_2_ powders and MAI powders are made as target source, and pre-heated to 116 °C and 325 °C, respectively, which has achieved the PSCs yield an PCE of 15.4%.^8^ This method fabricates high quality and uniformity of the perovskite films, subsequently resulting in good performance. But this method is very dependent on high temperature and high vacuum conditions. Alternate methods research in the literature^32^ is vapor–solid reaction (VSR), depositing the perovskite film with low temperature (Fig. 10(b)). First, the PbI_2_ film was spin-coated onto the ETL, and then baking on a 70 °C hot plate in air for 10 min. Second, MAI powders were dissolved in ethanol. Then the solution was homogeneously sprayed onto the bottom surface of the top plate that had been keeping at 80 °C. Finally, inside vacuum desiccator, two parallel hot plates (PHP) were putted together to synthesize perovskite thin films.^32^ H. Zhou and S. Yin *et al.*^32^ used this method to achieve the 8 × 8 cm^2^ PSCs module, the average PCE was 6.0% with the active area of 1.5 cm^2^.

### 3.3 Gas-induced method

**Fig. 11:**
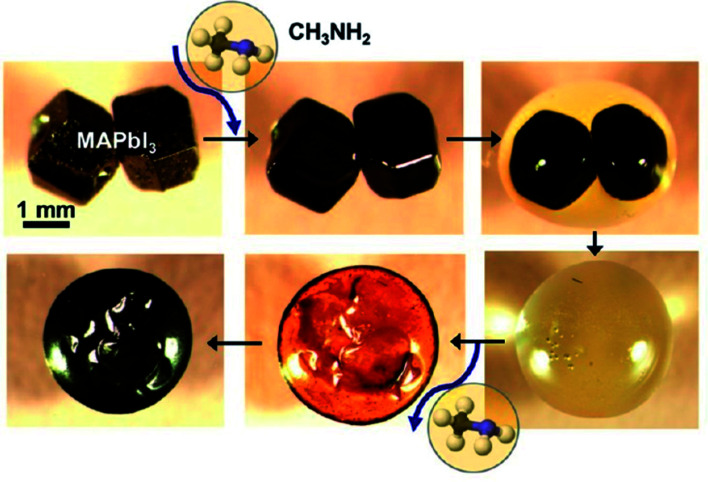
*In situ* optical microscopy of the morphology evolution of two touching MAPbI_3_ perovskite crystals (same magnification) upon exposure to CH_3_NH_2_ gas and CH_3_NH_2_ degassing.^93^

**Fig. 12:**
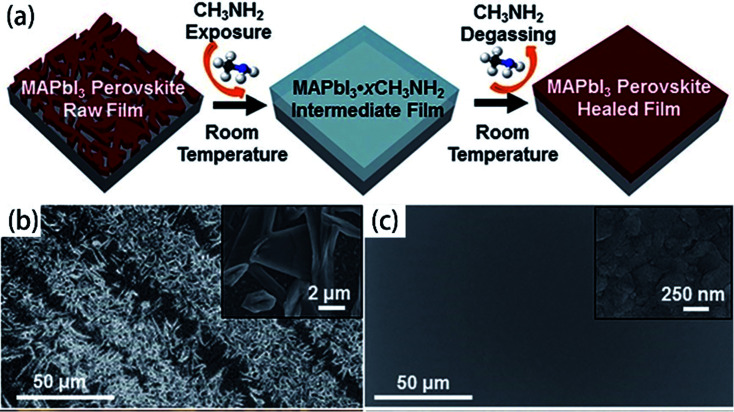
(a) Schematic illustration of MA induced defect-healing of MAPbI_3_ perovskite thin films. SEM images of MAPbI_3_ thin films: (b) raw film and (c) healed film.^93^

For the organic–inorganic halide perovskites (OIHPs) materials, gas-induce formation/transformation (GIFT) reveal surprising properties, such as gas-induced phase/morphology transformation.^92^ Z. Zhou, S. Pang, G. Cui *et al.*^93^ reported that the discovery of CH_3_NH_2_ (MA) induced phase/morphology transformation of the MAPbI_3_. As show in Fig. 11, MA gas is introduced at room temperature (RT), after 120 min, two MAPbI_3_ single-crystals become liquefied (MAPbI_3_·*x*CH_3_NH_2_), eventually, merge into one liquid sphere.^93^ Then MA gas is removed, after 120 min, perovskite back-conversion completed. Fig. 12(b) shows a poor quality of MAPbI_3_ thin film (incomplete coverage, rough), then the MA gas treatment has been introduced to create smooth, uniform and full coverage MAPbI_3_ thin films (Fig. 12(c)).^93^

**Fig. 13:**
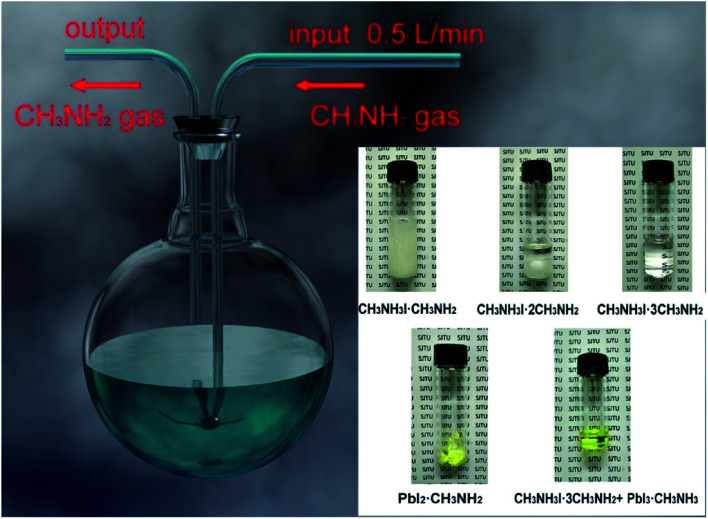
Diagram of the CH_3_NH_2_ introduced CH_3_NH_3_I and PbI_2_ powers and the mixture of CH_3_NH_3_I·3CH_3_NH_2_ and PbI_2_·CH_3_NH_2_.^36^

**Fig. 14:**
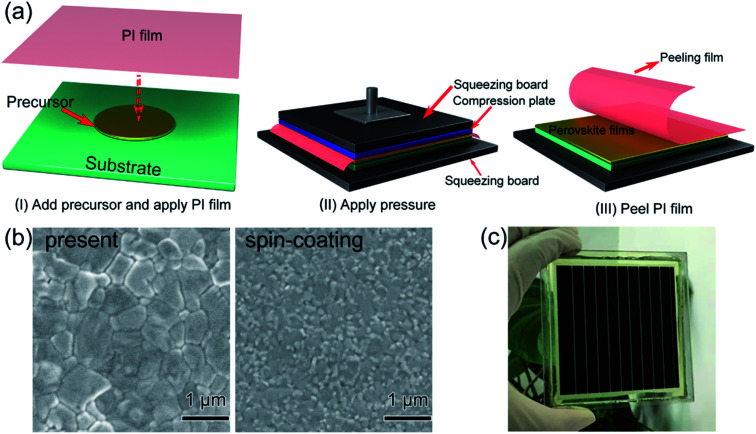
Diagram of the pressure processing method for the deposition of perovskite films. (a) The steps of the pressure processing method. (b) SEM images. (c) Photograph of a perovskite module.^36^

In 2017, M. Grätzel and L. Han *et al.*^36^ achieved 8 × 8 cm^2^ perovskite films *via* GIFT, briefly, at atmospheric environment, dried CH_3_NH_2_ gas (0.5 l min^−1^) was passed into a bottle that contained 2 mmol CH_3_NH_3_I or PbI_2_ powders (Fig. 13). After 30 min, the CH_3_NH_3_I powders changed into transparent colorless liquid (CH_3_NH_2_I·3CH_3_NH_2_), and the PbI_2_ powders changed into a pale-yellow paste (PbI_2_·CH_3_NH_2_, Fig. 13).^36^ For the synthesis of perovskite precursor, CH_3_NH_2_I·3CH_3_NH_2_ and PbI_2_·CH_3_NH_2_ were blended stoichiometrically and ultrasonicated for 15 min (Fig. 13).^36^ The perovskite precursor (200 μl) was dropped on a 8 × 8 cm^2^ substrate and then the precursor was covered by the polyimide (PI) film.^36^ A pressure of 120 bar was loaded *via* a pneumatically driven squeezing board which spread the liquid precursor under the PI film. The pressure was held for 60 s and then unloaded. The thin liquid film covered with the PI film was heated at 50 °C for 2 min before peeling off the PI film. After peeling the PI film (50 mm s^−1^), a dense and uniform perovskite film was formed (Fig. 14(b)).^36^ They achieved the PSCs with the device area 36 cm^2^ (Fig. 14(c)) that had a certified PCE of 12.1%.^36^

### 3.4 Other approaches

**Fig. 15:**
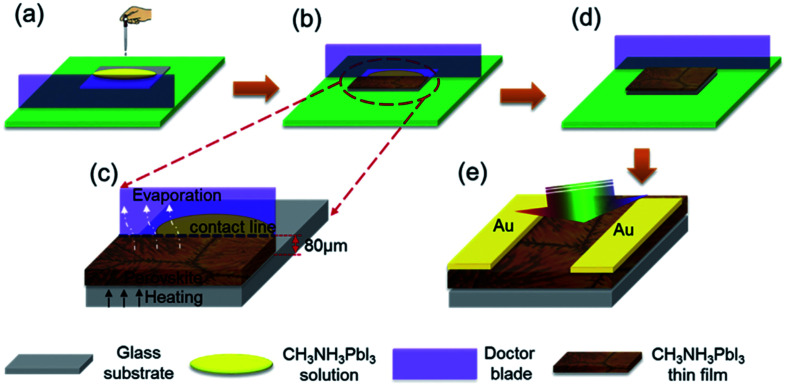
Schematic representation of *in situ* doctor blading technology for fabricating CH_3_NH_3_PbI_3_ films.^22^

**Fig. 16:**
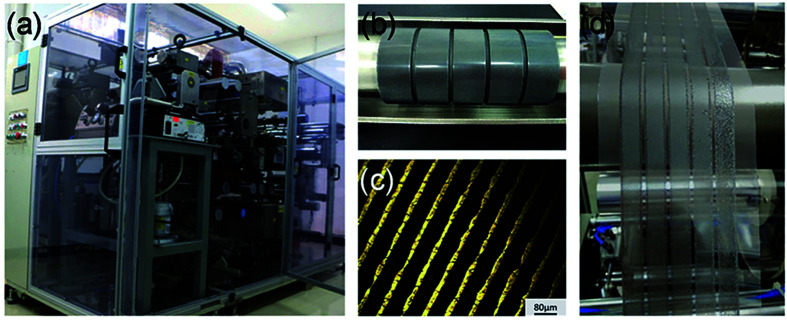
(a) Photo of self-developed R2R multi-function printer. (b) Photo of micro-gravure printer roller. (c) Optical microscope image of engraved micro-gravure printer roller. (d) A photo of R2R printing process.^94^

In addition, a blade coating technology is also frequently used, the schematic shows in Fig. 15.^22^ The advantage of the blade coating technology can control the distance between blade and the substrate, and the *in situ* thermal-treatment temperature. In 2015, S. Razza and A. D. Carlo *et al.*^47^ used the blade coating technology, which achieved a module PSCs with a 10.1 cm^2^ active area that had the efficiency of 10.4%.^47^ Meanwhile, an efficiency of 4.3% had been measured for a module area of 100 cm^2^.^47^ In 2016, J. L. Yang *et al.*^94^ reported an approach to fabricate ultra-long nanowires array and highly oriented CH_3_NH_3_PbI_3_ thin films in ambient environments, briefly, this approach included large-scale roll-to-roll micro-gravure printing and doctor blading (Fig. 16), which produced perovskite nanowires lengths as long as 15 mm.^94^

## 4. Methods of improving PCE for large-area (≥1 cm^2^) perovskite solar cells

For the large-area PSCs device, improving the PCE, the first method is to change the chemical composition of perovskite, adjusting its band gap and increasing the charge generation.^3,29,31,35,38,46^ The second approach is to increase the grain size of perovskite, decreasing the cracks and pinholes, that reduces the bulk defect recombination and electric leakage, and increase *V*_oc_.^35–38^ The third approach is interface modification, which reduces interface contact resistance, and reduce interface and surface recombination, and increase *J*_sc_.^31,41,45,95^

**Fig. 17:**
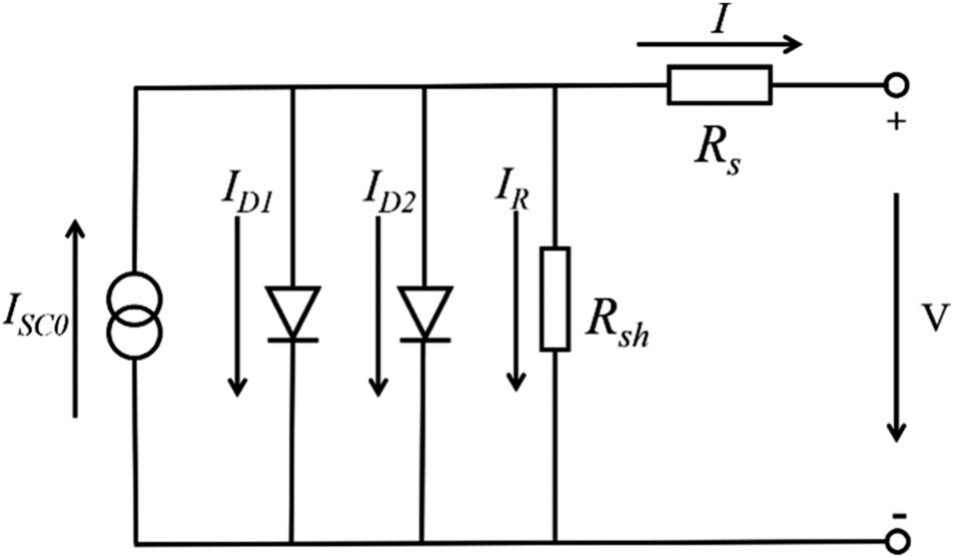
Incorporating these resistances into the circuit model.^96^

For the large-area PSCs device, with the increasing of cell size, the series resistance (*R*_s_) increase among the charge transfer layers, the absorber layer and the electrode layers. At the same time, the number of the crack and the pinholes increase, that from the shunt resistance (*R*_sh_) and the value of *R*_sh_ decrease. Incorporating these resistances into the circuit model of the solar cells device shows in Fig. 17.^96^ The increasing of *R*_s_ and the decreasing of *R*_sh_ increase the interface losses of the large-area PSCs device, that is the major reason of the lower efficiency for the large-area PSCs device.^95^

**Fig. 18:**
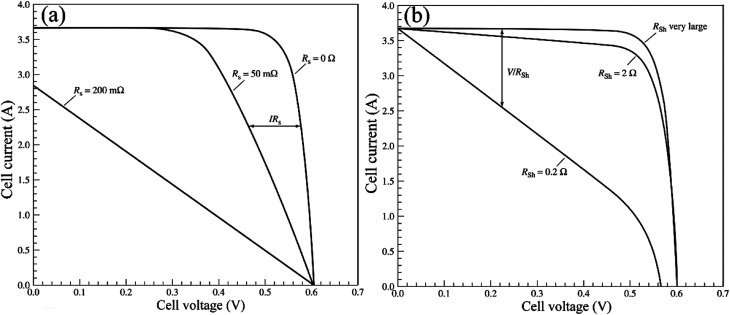
(a) *I*–*V* curve of *R*_s_, (b) *I*–*V* curve of *R*_sh_.^96^

The current expression in the circuit can be written as eqn (2).^96^2
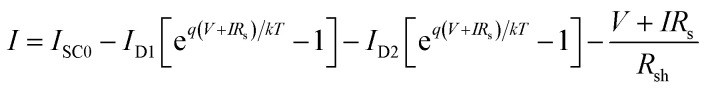
where *I*_SC0_ is the short-circuit current when there are no parasitic resistances (*R*_s_ and *R*_sh_). The effect of these parasitic resistances on the *I*–*V* characteristic is shown in Fig. 18. Form the eqn (2), the series resistance, *R*_s_ increase, has no effect on the open-circuit voltage, but reduces the short-circuit current (*J*_sc_) and fill factor (FF) (Fig. 18(a)). Conversely, the shunt resistance, *R*_sh_ decrease, has no effect on the short circuit current, but reduces the open-circuit voltage (*V*_oc_) and FF (Fig. 18(b)).

### 4.1 Chemical molecular engineering

For the perovskite material, its band gap can regulate *via* exchange the chemical molecular or element, achieving an ideal band gap of the perovskite material. Such as, through intramolecular exchange, formamidinium (FA) molecular is frequently used to replace methylamine (MA) in MAPbI_3_, forming FAPbI_3_ and adjusting the band gap. In 2015, W. S. Yang and J. H. Noh *et al.*^29^ have fabricated FAPbI_3_ films, its band gap is 1.47 eV smaller than MAPbI_3_ (1.50 eV). Meanwhile, the PCE of FAPbI_3_-based PSCs is up to 20.1%. In 2017, J. Y. Kim and D. S. Kim *et al.*^35^ fabricated (FAPbI_3_)_0.85_(MAPbBr_3_)_0.15_ (1.55 eV) as absorber layer of the PSCs device, which had a maximum PCE exceeding 18% over a 1 cm^2^ active area. In 2017, E. K. Kim, J. H. Noh and S. I. Seok *et al.*^3^ introduced additional iodide ions into the organic cation solution, that decreased the concentration of deep-level defects. They fabricated of the (FAPbI_3_)_*x*_(MAPbBr_3_)_1−*x*_-based PSCs with a certified PCE of 19.7% in 1 cm^2^ cells.^3^ Adding inorganic cesium to triple-cation perovskite compositions, E. H. Sargent *et al.*^31^ have reported the best-performance large-area (1.1 cm^2^) PSCs (Cs_0.05_FA_0.81_MA_0.14_PbI_2.55_Br_0.45_, 1.60 eV), that has a PCE of 20.3%. In 2017, X. W. Zhang and J. B. You *et al.*^38^ used the (FAPbI_3_)_1−*x*_(MAPbBr_3_)_*x*_ (1.55 eV) as absorber layer for PSCs with the certified efficiency of 20.1% in large-area (1 cm^2^).^38^

### 4.2 Improving preparation technology

**Fig. 19:**
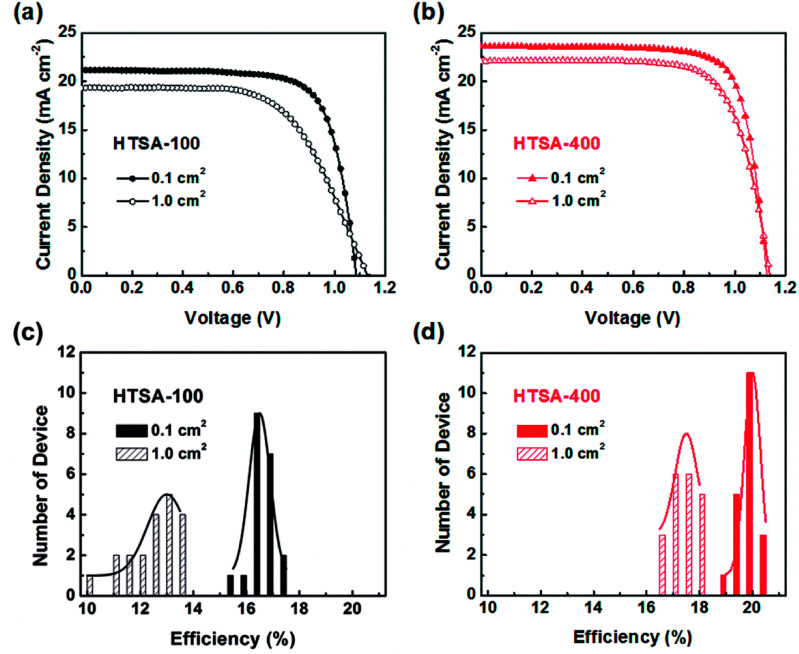
(a) Current−voltage curves of perovskite solar cells derived from HTSA-100 and (b) HTSA-400 with an active area 0.1 cm^2^ and a 1 cm^2^. (c) Histogram of PCEs derived from HTSA-100 and (d) HTSA-400 with an active area 0.1 cm^2^ and 1 cm^2^.^35^

The high quality (cracks- and pinholes-free) large-area perovskite film is precondition for the achieving high PCE of PSCs. Because the cracks and pinholes can form electric leakage (forming the *R*_sh_), which lead to the decreasing the *V*_oc_ and FF, and reduce the PCE of PSCs. So, M. J. Kim and G. H. Kim *et al.*^35^ developed one step spin-coating, and using high-temperature short-time annealing process (Fig. 7(a)), achieving the perovskite grains with sizes more than 1 μm without pinhole (HTSA-400, Fig. 7(d, e, h and i)). They fabricated PSCs device with 1 cm^2^, which achieved the PCE of 18.32% with HTSA-400 (Fig. 19(d)), but the PCE is only 13.82% with HTSA-100 (Fig. 19(c)).^35^ X. Li and M. Grätzel *et al.*^37^ used the vacuum flash-assisted solution processing (VASP) to fabricate perovskite film (Fig. 8(a)), the sizes of perovskite grains were between 400 and 1000 nm (Fig. 8(c)). They fabricated the PSCs device with an aperture area exceeding 1 cm^2^, the certified PCE of 19.6%.^37^ In 2015, Z. Zhou, S. Pang, G. Cui *et al.*^93^ reported that the MA gas treatment has been introduced to create smooth, uniform and full coverage MAPbI_3_ thin films (Fig. 12(c)).^93^ This MAPbI_3_ was used to fabricate the PSCs device, the PCE increased from 5.7% to 15.1%, was observed, which was clearly the result of the improving film morphology.^93^

### 4.3 Interface engineering

Interface engineering can optimize interface contact, mitigate carrier recombination and increase carrier collection, which is extremely important to achieve high-performance and high-stability PSCs. Interface engineering includes doped, plasma etching, self-assembled monolayers and interface buffer layer *etc.*

**Fig. 20:**
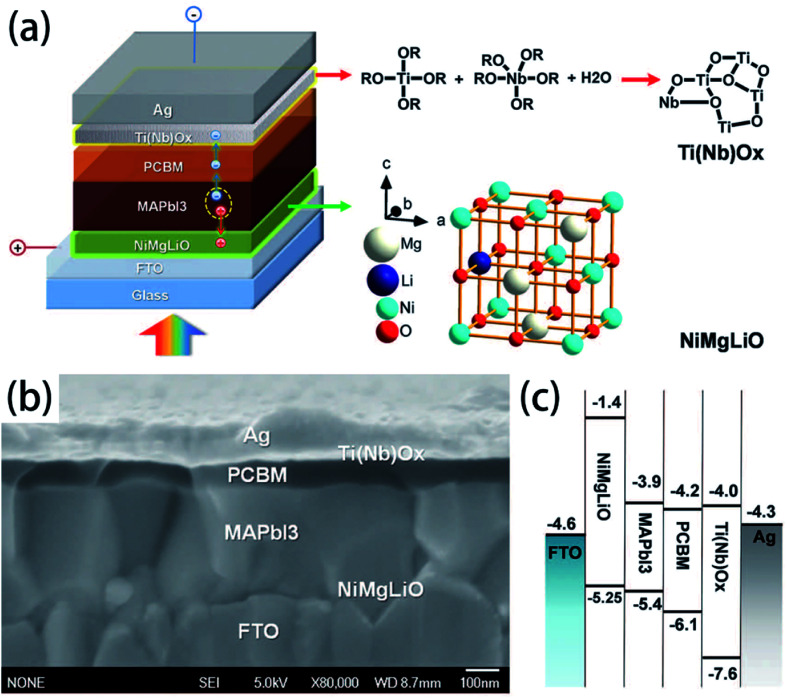
Structure and band alignments of the PSCs, (a) diagram of the cell configuration highlighting the doped charge carrier extraction layers. (b) A high-resolution cross-sectional SEM image of a complete solar cell. (c) Band alignments of the solar cell.^41^

**Fig. 21:**
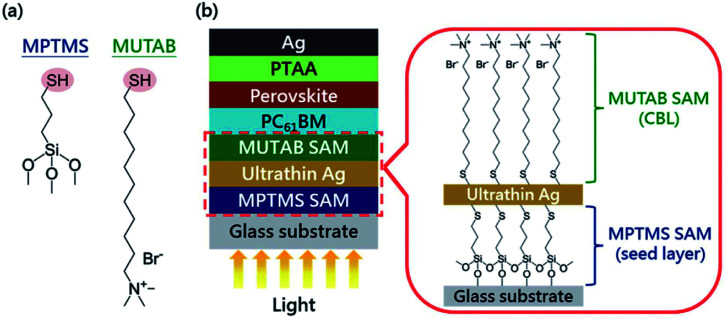
(a) Chemical structures of SAM molecules. (b) Schematic illustration of the device architecture used in this study.^45^

Doping for the charge transport layers, that can improve their electrical performance, such as improving carrier concentration and mobility. For Li–Mg co-doped NiO films, the conductivity is 2.32 × 10^−3^ S cm^−1^, ∼12 times greater than that of the pure Mg_*x*_Ni_1−*x*_O.^41^ The conductivity of Nb^5+^ doped TiO_2_ films is ∼10^4^ S cm^−1^, ∼100 to 1000 times greater than that of the pure TiO_2_.^41^ In 2015, M. Grätzel and L. Y. Han *et al.*^41^ have used Mg–Li co-doped NiO as HTL and Nb doped TiO_*x*_ as ETL material in inverted planar PSCs to achieve very rapid carrier extraction, increasing the cell FF from 0.64 to 0.827. Meanwhile, they fabricated a large-area (>1 cm^2^) PSCs (Fig. 20(a)) with a certified efficiency of 15%.^41^ The contact-passivation can mitigate interfacial recombination and improve interface binding in low-temperature planar PSCs. H. R. Tan and E. H. Sargent *et al.*^31^ reported a contact-passivation strategy using chlorine-capped TiO_2_ (Cl–TiO_2_) colloidal nanocrystal film as ETL, the charge-recombination lifetime increased from 64 μs to 145 μs compare with pure TiO_2_ film.^31^ They fabricated the planar PSCs for active areas of 1.1 cm^2^, that achieved a certified efficiency of 19.5% without hysteresis.^31^ Interlayers are thin layers or monolayers of organic molecules that modify a specific interface in the solar cell.^97^ In 2016, C.Y. Chang and Y. C. Chang *et al.*^45^ reported an approach for the modification of interface layer *via* introducing thiol-functionalized self-assembled monolayers (SAMs, Fig. 21(b)), which decreased interface charge recombination and increased the value of *J*_sc_ (19.43 mA cm^−2^ to 21.68 mA cm^−2^) and FF (0.67 to 0.72). They fabricated a large-area (1.2 cm^2^) PSCs with the PCE up to 15.98%.^45^ Y. Wu and X. Yang *et al.*^42^ reported a perovskite–fullerene graded heterojunction structure, which improved the photoelectron collection and reduced recombination loss. They fabricated the PSCs of 1.022 cm^2^, that had a certified PCE of 18.21%.^42^

## 5. Stability of large-area (≥1 cm^2^) perovskite solar cells

In recent years, the certificated PCE of the large-area (1 cm^2^) PSCs has achieved 20.1%.^38^ However, the major issue of large-area PSCs for commercial applications is the poor long-term device stability. For the stability of the perovskite materials and devices, it is necessary to consider the effects of temperature, illumination and ambient (oxygen, moisture) exposure. Many papers have reported about this important issue.^1,51,53,97–107^

### 5.1 Degradation mechanisms

**Fig. 22:**
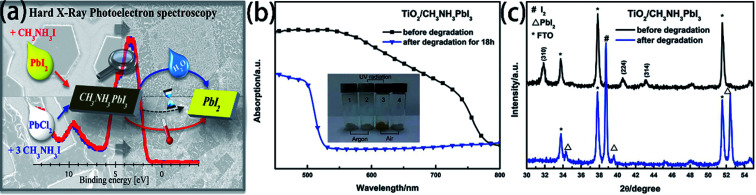
(a) Degradation of MAPbI_3_ in moisture and air atmosphere.^109^ (b) UV-vis absorption spectra of TiO_2_/MAPbI_3_ film before and after degradation.^48^ (c) XRD patterns of TiO_2_/MAPbI_3_ film before and after degradation.^48^

The degradation of the PSCs device includes the degradation of the active layer, the degradation of charge transport layers, and the degradation of electrodes.^104^ The MAPI_3_ films are frequently used as absorber layer film. But the major problem with MAPbI_3_ is that has thermal decomposition (exceeding 85 °C)^108,109^ and water decomposition.^1,48,110^ Some researchers have reported the decomposition process of MAPbI_3_. B. Philippe and H. Rensmo *et al.*^109^ exposed the MAPbI_3_ and MAPbI_3−*x*_Cl_*x*_ to various environments. From the photoelectron spectroscopy results with the different environments, the perovskite has decomposed into PbI_2_, but this degradation seems to occur already at 100 °C and is not only related to large humidity (Fig. 22(a)). Meanwhile, they observed a slow degradation occurs even when stored in an inert atmosphere such as argon.^109^ L. D. Wang *et al.*^48^ verified that oxygen, together with moisture, could lead to the irreversible degradation of MAPbI_3_. They exposed TiO_2_/CH_3_NH_3_PbI_3_ film to air with a humidity of 60% at 35 °C for 18 h, and then, the absorption between 530 and 800 nm greatly decreased (Fig. 22(b)), the MAPbI_3_ decomposed into PbI_2_ and I_2_ (Fig. 21(c)).^48^ The degradation mechanism of MAPbI_3_ upon exposure to moisture in absence of illumination involves the formation of hydrate form, which can be reversible.^11,48,111,112^ However, continuing exposure to moisture and/or exposure to illumination leads to the irreversible degradation to PbI_2_.^111^ For ETL material, TiO_2_ is especially sensitive to ultraviolet light, in the ultraviolet light, Ti^4+^ adsorb O_2_ and convert into Ti^3+^, increasing the charge recombination.^113^ Meanwhile, the lithium salt in spiro-MeOTAD is easy to absorb moisture and decrease the PSCs device stability.

### 5.2 Methods of improving stability

**Fig. 23:**
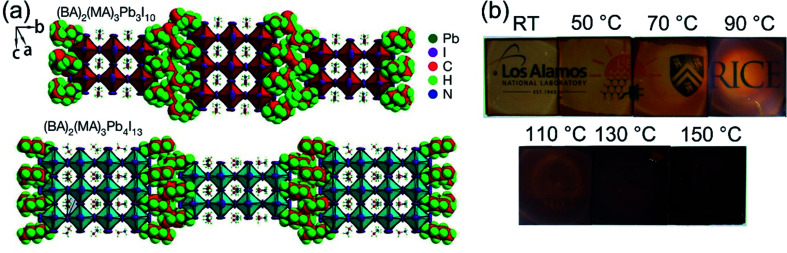
Crystal structure and thin-film characterization of layered perovskites. (a) The crystal structure of the Ruddlesden–Popper (BA)_2_(MA)_2_Pb_3_I_10_ and (BA)_2_(MA)_3_Pb_4_I_13_ layered perovskites. (b) Photos of (BA)_2_(MA)_3_Pb_4_I_13_ thin films cast from room temperature (RT) to 150 °C.^115^

**Fig. 24:**
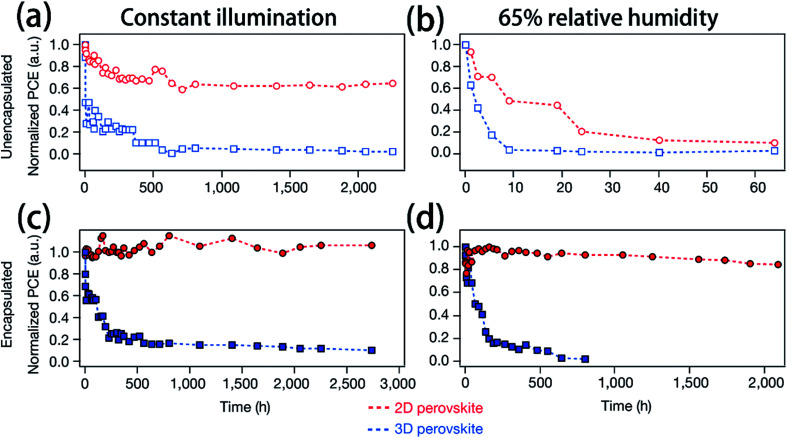
(a and c) Photostability tests under constant AM1.5G illumination for 2D ((BA)_2_(MA)_3_Pb_4_I_13_; red) and 3D (MAPbI_3_; blue) perovskite devices. (b and d) Humidity stability tests under 65% relative humidity at in a humidity chamber for 2D ((BA)_2_(MA)_3_Pb4I_13_; red) and 3D (MAPbI_3_; blue) perovskite devices.^115^

In recent years, many methods have been researched to improve the PSCs device stability. Due to the poor stability of MAPI_3_, the first method is to modify the chemical constituents or structure of the perovskite. For example, 2D perovskites, compared with 3D perovskites, 2D perovskites have the higher carrier mobility while maintaining good ambient stability.^114,115^ The 2D Ruddlesden–Popper layered perovskites ((BA)_2_(MA)_2_Pb_3_I_10_ and (BA)_2_(MA)_3_Pb_4_I_13_) have been studied (Fig. 23(a)).^115^ The (BA)_2_(MA)_3_Pb_4_I_13_ film color gets darker with increasing temperature (Fig. 23(b)).^115^ H. Tsai and W. Nie *et al.*^115^ have achieved a PCE of 12.51% with 2D (BA)_2_(MA)_3_Pb_4_I_13_ PSCs device. Under the constant light illumination, after 2500 h, the 2D perovskite devices is retaining 70% of its original PCE without encapsulated and 98% with encapsulated. The 3D perovskite devices have degraded < 10% of its original PCE after 2500 h (Fig. 24(a and c)). Fig. 24(b) shows the PCE of the unencapsulated 2D and 3D devices, that shows degradation after 60 h, under 65% relative humidity.^115^ With simple encapsulation, after 2500 h, the 2D devices retained 80% of its original PCE under 65% relative humidity, but the 3D devices had been degraded (Fig. 24(d)).^115^ K. Yao *et al.*^46^ used the polyethylenimine (PEI) cations to fabricate the 2D perovskite compounds (PEI)_2_(MA)_*n*−1_Pb_*n*_I_3*n*+1_ (*n* = 3, 5, 7), which was used as absorber layer to fabricate PSCs with an aperture area of 2.32 cm^2^ under ambient humidity that have a PCE up to 8.77%. After 500 h, the PCE of the 2D large-area PSCs device only decreased by ∼5%.^46^

**Fig. 25:**
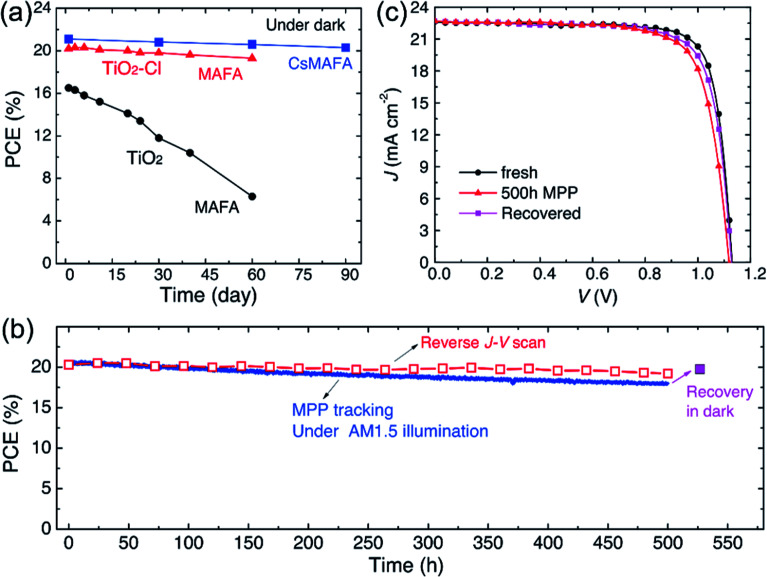
Long-term device stability of PSCs with TiO_2_–Cl and TiO_2_. (a) Dark storage stability of non-encapsulated PSCs. (b) Continuous maximum power point tracking for 500 hours of a high performance unsealed CsMAFA cell with TiO_2_–Cl in nitrogen atmosphere under constant simulated solar illumination. (c) *J*–*V* curves of the PSCs (CsMAFA) from (b) at various stages.^31^

**Fig. 26:**
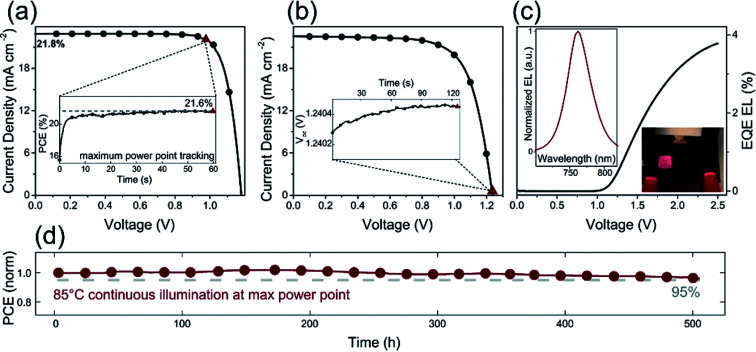
(a) *J*–*V* curve of RbCsMAFA solar cell. (b) *J*–*V* curve of the highest-*V*_oc_ device. (c) EQE electroluminescence (EL) as a function of voltage. (d) Thermal stability test of a perovskite solar cell.^116^

Furthermore, the alkali metal cation is introduced into the perovskite material, which can improve the stability of the PSCs device.^31,68,116^ E. H. Sargent *et al.*^31^ added cesium cation to fabricate a triple-cation perovskite compositions films (Cs_0.05_FA_0.81_MA_0.14_PbI_2.55_Br_0.45_), that was made the large-area (1.1 cm^2^) PSCs with a PCE up to 20.3% (Fig. 25(b and c)). After 90 days, the PSCs devices retained 96% of its initial PCE (Fig. 25(a)).^31^ Rubidium (Rb) cations can stabilize the black phase of FA perovskite and be integrated into PSCs, M. Saliba and M. Grätzel *et al.*^116^ have used RbCsMAFAPbI_3_ as absorber layer of the PSCs device. After 500 h at 85 °C under continuous illumination, the device has retained 95% of its initial PCE (Fig. 26(d)).^116^

The second method for improving the PSCs device stability is to modify the charge transport layer (ETL and HTL), or use the new type charge transport material.^61^ Because TiO_2_ is especially sensitive to ultraviolet light,^113^ some new ETL materials have been reported. A. D. Carlo *et al.*^117^ reported an additional lithium-neutralized graphene oxide (GO-Li) layer as interface layer was inserted between TiO_2_ ETL and perovskite layer, that improved the stability of PSCs devices.^117^ A. Hagfeldt *et al.*^118^ has used ZnO nanorod arrays as ETL replace the TiO_2_, achieving the PSCs device, it has been exposed in atmospheric environment without encapsulation, and maintaining 90% of the original efficiency. X. W. Zhang and J. B. You *et al.*^57^ have used SnO_2_ as ETL for planar-structure PSCs, it is found that the devices can maintain almost their original efficiency when store in dry air conditions for 40 days.^57^ J. H. Noh and S. I. Seok *et al.*^66^ used La-doped BaSnO_3_ as ETL, the PSCs retained 93.3% of its initial PCE after 1000 hours, whereas the TiO_2_ cells had completely degraded within 500 hours.

For the HTL materials, spiro-OMeTAD is the most commonly used HTL material,^35,57,67^ the certified PCE of 22.1% in small cell.^3^ But the lithium salt in spiro-MeOTAD is easy to absorb moisture and reduce the PSCs device stability. So inorganic and hydrophobic hole transport material are used to improve the PSCs device stability.^41,54,74,78,79^ M. Grätzel and L. Y. Han *et al.*^41^ used Li_0.05_Mg_0.15_Ni_0.8_O as HTL material and Ti(Nb)O_*x*_ as ETL to fabricate inverted planar heterojunction structure device (p–i–n), under simulated solar light, the PSCs device maintained 90% of the original efficiency after 1000 h. S. H. Yang *et al.*^79^ has fabricated inverted planar heterojunction structure for NiO-based PSCs device (p–i–n), achieving more than 85% of its original PCE has been kept after 150 days. Z. B. He *et al.*^78^ used NiO_*x*_ nanocrystal as HTL in planar PSCs device. After 1000 h, the PCE of PSCs device maintained 87% of its initial value. N. Arora and M. Grätzel *et al.*^74^ used one new HTL material CuSCN. They achieved the PSCs with PCE > 20%, after 1000 hours at 60 °C, the PSCs devices retained >95% of their initial efficiency. CuGaO_2_ as HTL in n–i–p configuration PSCs, exposing it directly to the ambient environment without encapsulation. After 30 days, it maintains 87% its initial PCE.^54^

**Fig. 27:**
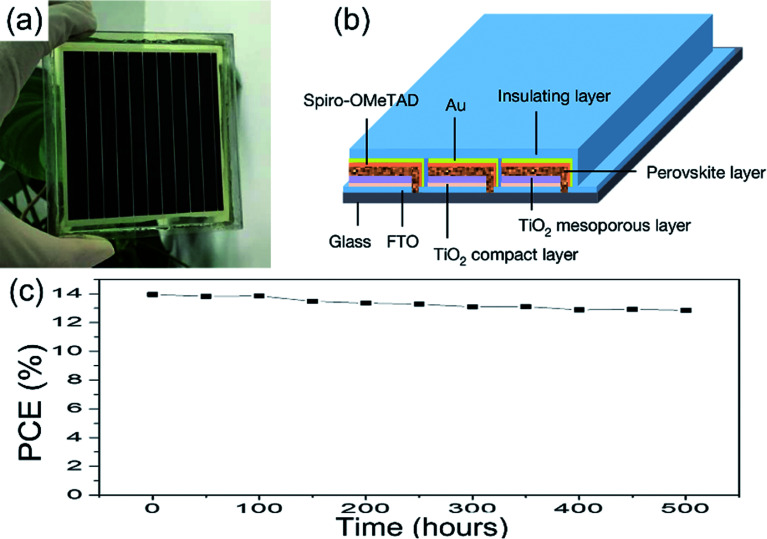
Illustration of the perovskite module and device performance. (a) Photograph of a module. (b) Diagram of the module structure. (c) Evolution of the photovoltaic stability of an encapsulated perovskite solar module.^36^

Other methods for improving the PSCs device stability include the PSCs structure optimization, interface optimization, encapsulation, *etc.*^81,82,119^ A hole-conductor-free structure of the PSCs can achieve long-term stability. Exposing the PSCs device (c-TiO_2_/m-TiO_2_/ZrO_2_/carbon) under full AM 1.5 simulated sunlight over 1008 hours, the PCE maintains 100% of its initial value.^82^ To improve the stability of the device, the insulation material encapsulate the PSCs device is frequently used. M. Grätzel and L. Y. Han *et al.*^36^ encapsulated the large-area PSCs device (36.1 cm^2^, TiO_2_ ETL, Fig. 27(a and b)) by the insulation material, the module retained 90% of its initial performance after 500 h (Fig. 27(c)).

## 6. Other issues

### 6.1 Cost analysis

For conventional solar PV technology, it need high energy and vacuum to process solar cells. Thus, these PSCs can turn-out to be a promising solution in replacing the conventional PV technology. In this section, we briefly analyze the cost for various raw materials of a 1 m^2^ PSCs module. Conventional PSCs device architecture is shown in Fig. 4(a), that include glass substrate, TCO (FTO), ETL (TiO_2_), perovskite absorber layer (MAPbI_3_), HTL (spiro-OMeTAD) and metal electrode (Au). For 1 m^2^ conventional PSCs module, raw material utilization for cleaning, deposition of various layers and encapsulation of the module were extracted from various available literature sources and their corresponding data are included in Table 3.^120,121^ From the data (Table 3), it is clear that about 43% of the total raw material cost is from FTO substrate, about 34% from the HTL material (spiro-OMeTAD), and 18% from metal electrode (Au).^120,121^

**Table 3:** Cost breakdown of raw materials utilized in fabricating a PSC module of 1 m^2^ with 70% active area^120,121^

Raw material	Qty	Price (in USD)	Contribution towards total cost (%)	Comments
FTO glass	1 m^2^	766	43.0500	Processing cost of FTO is included
Ethanol	32.84 ml	4.59		
DI water	32.70 ml	0.002		
HCL solution	4.66 ml	1.8204		

**Blocking layer**				
TAA	19.16 ml	7.339	0.0066	For 100 nm layer and post deposition cleaning is included
Ethanol	32.84 ml	4.59		
DI water	32.70 ml	0.002		

**ETL material**				
TiO_2_	49.5 g	41.12	0.02291	For 250 nm thick layer

**Perovskite layer**				
PbI_2_	1.38 g	4.26	0.003811	For 100 nm thick solvent PbI_2_ followed by 200 nm thick solvent MAI
DMF	2.98 ml	0.5502		
MAI	0.143 g	0.49		
IPA	14.29 ml	1.538		

**HTL material**				
Spiro-OMeTAD	0.850 g	603.65	33.973	For 200 nm thick solvent HTM layer
Chlorobenzene	10.67 ml	5.9		

**Cathode**				
Au	1.65 g	330	18.392	For 100 nm thick layer

**Encapsulation**				
3 M tape		22.32	0.012	Taped on both sides
PET	61.7 g	0.03		

These data suggest the need for replacement of conventional FTO substrate, HTL material and Au electrode. The efficiency of the PSCs device on ITO-free analogues achieved 11%.^122^ Some new and cheap HTL materials have been reported, such as NiO (PCE ∼ 18.47%),^79^ triazine-Th-OMeTPA (PCE ∼ 12.51%),^75^ CuGaO_2_ (PCE ∼ 18.51%),^54^ CuSCN (PCE ∼ 20.4%),^74^ NiMgLiO (PCE ∼ 16.2%),^41^*etc.* Meanwhile, the efficiency for carbon based HTM-free PSCs devices achieved 15.9%.^81^ Although, the PCE of spiro-OMeTAD-free PSCs device is little lower than the conventional PSCs, with small sacrifice in efficiency, low-cost and highly stable carbon based HTM-free PSCs can be fabricated.

### 6.2 Environmental issues – the presence of lead

Environmental issues are a well-recognized issue for PSCs.^104,123^ Like CdTe, a toxic heavy metal exists in the PSCs devices. But, the CdTe is very chemically stable, organolead halide perovskites are not stable and upon ambient exposure they can degrade into products that are readily leached into the environment.^104,123^ In the life cycle assessments (LCA), the hazards of Pb for environmental impacts exist in all stages, which include raw material extraction, synthesis of starting products, fabrication, use and decommissioning.^120^ Thus, ideally PSCs should be subject to even more stringent safety standards and any commercial products should have clear plans for end-of-life disposal and/or recycling.^104,123^

To address the concerns about lead, lead-free perovskite materials have attracted the attention of many researchers, which include tin-based perovskite materials and other perovskites materials (lead-free and tin-free perovskites, such as MA_2_CuCl_*x*_Br_4−*x*_,^124^ CsGeI_3_, MAGeI_3_, and FAGeI_3_,^125^ A_3_Sb_2_I_9_ (A = Cs, Rb),^126^ Cs_2_BiAgCl_6_,^127^ (*N*-methylpyrrolidinium)_3_Sb_2_Br_9_,^128^*etc.*). But, compare with lead-based perovskites, the efficiencies of tin-based PSCs commonly well below 10%,^129,130^ the PCE values for other perovskites have been below 1%.^124–126^

Thus, improving encapsulation technologies, it could limit the Pb leakage during the cell operation. Researching the lead-free perovskite materials, achieving high performance lead-free PSCs device, which could to replace the lead-based PSCs device.

## 7. Conclusions

In this article, we briefly summarized the studies on large-area PSCs in recent years. Progress has been made in manufacturing larger area cells as well as modules, which is the interesting for commercialization of the technology. Approaches for fabricating the lager-area perovskite film layer are described such as spin-coating, vapor deposition, gas-induced and blade coating *etc.* It is demonstrated that these processes are useful to realize more uniform perovskite layer with larger grain sized and better surface coverage, which strongly affect consequent photovoltaic performance of devices.

Going forward, PSCs will have to reduce non-radiative recombination and improve charge transport in order to achieve the highest possible *V*_oc_ values and fill factors. For the large-area PSCs device, improving the PCE, the first method is to change the chemical composition of perovskite, adjusting its band gap and increasing the charge generation. The second approach is to increase the grain size of perovskite, decreasing the cracks and pinholes, that reduces the bulk defect recombination and electric leakage, and increase *V*_oc_. The third approach is interface modification, which reduces interface contact resistance, and reduce interface and surface recombination, and increase *J*_sc_. Meanwhile, one key issue of the large-area PSCs is the long-term poor stability. To the improving of the stability of PSCs, which requires interdisciplinary research to find new stable materials, the choice of electrodes, barrier layers, charge transport layers and encapsulation strategies. Undoubtedly, in the near future, halide perovskite materials have emerged as an attractive alternative to conventional silicon solar cells.

## Conflicts of interest

The authors declare that there is no conflict of interests regarding the publication of this paper.

## Supplementary Material
